# Supplementary material for: Rapid Detection of Predominant SARS-CoV-2 Variants Using Multiplex High-Resolution Melting Analysis
Source: Microbiol Spectr. 2023 May 16;11(3):e00055-23. doi: 10.1128/spectrum.00055-23 (PMC10269585; doi:10.1128/spectrum.00055-23)
Supplement: Supplemental file 1 — Supplemental material. Download spectrum.00055-23-s0001.docx, DOCX file, 0.18 MB [file spectrum.00055-23-s0001.docx]

***Supplementary Material***

**Rapid Detection of Predominant SARS-CoV-2 Variants Using Multiplex High-Resolution Melting Analysis**

Liying Sun^a,b#^, Liqin Wang^a,b#^, Chi Zhang ^a,b^, Yan Xiao^a,b,c^, Lulu Zhang^a,b^, Ziyuan Zhao^a,b^, Lili Ren ^a,b,c^*, Junping Peng ^a,b^*

^a^ NHC Key Laboratory of Systems Biology of Pathogens, Institute of Pathogen Biology, Chinese Academy of Medical Sciences & Peking Union Medical College, Beijing, China

^b^ Key Laboratory of Respiratory Disease Pathogenomics, Chinese Academy of Medical Sciences and Peking Union Medical College, Beijing, China

^c^ Christophe Merieux Laboratory, Institute of Pathogen Biology, Chinese Academy of Medical Sciences and Peking Union Medical College, Beijing, China.

^#^ These authors contributed equally in this study.

Running Head: Multiplex High-Resolution Melting SARS-CoV-2 Assay

*Corresponding author: Junping Peng and Lili Ren

Junping Peng

E-mail: pengjp@hotmail.com.

Mailing address: No. 6 Rongjing Eastern Street, BDA, Beijing 100176, China.

Lili Ren

E-mail: renliliipb@163.com.

Mailing address: No.9 Dong Dan San Tiao, Dongcheng District, Beijing 100730, P. R. China.

**Supplementary information**

**Supplemental Table S1.** The primer pairs of PCR for conventional sequencing.

**Supplemental Figure S1.** The melt curve of the assay 3 for gradient diluted sample.

Supplemental Table S1. The primer pairs of PCR for conventional sequencing.

| Targets | Primer | Sequence (5’-3’) |
| --- | --- | --- |
| S | S-sanger-F1 | TTGTTATTTCTAGTGATGTTCTTGTTAAC |
|  | S-sanger-R1 | GAAGAATCACCAGGAGTCAAATAA |
|  | S-sanger-F2 | CCTCAGGGTTTTTCGGCT |
|  | S-sanger-R2 | GGTTTGAGATTAGACTTCCTAAACAA |
|  | S-sanger-F3 | CTGATTATAATTATAAATTACCAGATGATTTTACA |
|  | S-sanger-R3 | AATTAGTCTGAGTCTGATAACTAGCG |
|  | S-sanger-F4 | TTCAAACACGTGCAGGCTG |
|  | S-sanger-R4 | AAGGTCCAACCAGAAGTGATT |
|  | S-sanger-F5 | CTTTGCTCACAGATGAAATGATTG |
|  | S-sanger-R5 | TGCTGACTGAGGGAAGGAC |
|  | S-snager-F6 | TCTGCTAATCTTGCTGCTACTAA |
|  | S-sanger-R6 | CAATTGTGAAGATTCTCATAAACAAATC |


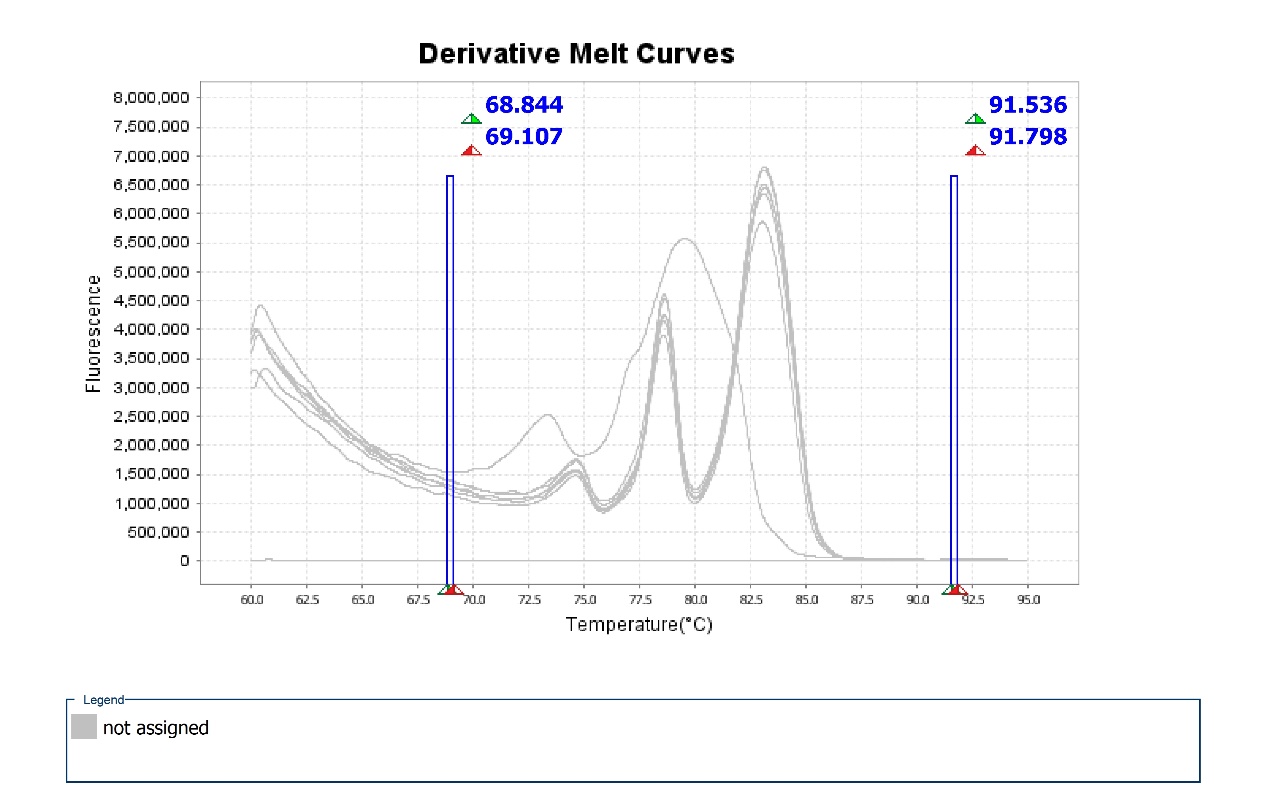
Figure S1. The melt curve of the assay 3 for gradient diluted sample.
